# Coffee Consumption and Pancreatic Cancer Risk: An Update Meta-analysis of Cohort Studies

**DOI:** 10.12669/pjms.321.8761

**Published:** 2016

**Authors:** Heng-Quan Ran, Jun-Zhou Wang, Chang-Qin Sun

**Affiliations:** 1 Dr. Heng-Quan Ran, MD, Division of Hepatobiliary Pancreatic Surgery, Panzhihua Central Hospital, Sichuan Province, China; 2 Dr. Jun-Zhou Wang, MD, Division of Hepatobiliary Pancreatic Surgery, Panzhihua Central Hospital, Sichuan Province, China; 3 Dr. Chang-Qin Sun, MD, Division of Hepatobiliary Pancreatic Surgery, Panzhihua Central Hospital, Sichuan Province, China

**Keywords:** Coffee, Pancreatic cancer, Meta-analysis

## Abstract

**Background & Objective:**

The results of epidemiologic studies on the relationship between the coffee consumption and pancreatic cancer risk were inconsistent. Thus, we performed an update meta-analysis of cohort studies to quantitatively summarize the association between coffee consumption and pancreatic cancer risk.

**Methods:**

We searched CBM (China Biology Medicine disc) and MEDLINE for studies of coffee consumption and pancreatic cancer risk up to June 2015. A total of 20 cohort studies were identified in this meta-analysis, and we analyzed these studies using random effects model. The dose-response analysis was conducted too.

**Results:**

The overall relative risk (RR) for highest coffee consumption *versus* lowest coffee consumption was 0.75 (95% Confidence Interval (CI), 0.63-0.86). Statistic significant heterogeneity was found among these studies (*I*^2^ =37.8%, *P* for heterogeneity =0.045). The pooled RR for increment of 1 cup/day of coffee consumption was 0.99 (95%CI, 0.96-1.03) for the nine studies, without statistically significant.

**Conclusions:**

High coffee consumption is associated with a reduced pancreatic cancer risk. However, the result should be accepted with caution, due to the potential confounder and bias could not be excluded. Further well designed studies are needed to confirm the finding.

## INTRODUCTION

Pancreatic cancer is the eighth most common cause of death from cancer worldwide.^1^ About 46 420 new pancreatic cancer cases were diagnosed and 39 590 people died from this cancer in the United States in 2014.^2^ Although the diagnosis and treatment of pancreatic cancer has been evident improvement, the five-year survival rate for the disease is still no more than 5%.^3^ Thus, it is very important to detect modifiable risk factors that may develop into the primary prevention for this cancer.

Coffee is one of the most popular beverages over the world. Since the early 1980s, epidemiologic studies on the relationship between the coffee consumption and pancreatic cancer risk have been conducted in different countries,^4^-^21^ and two meta-analysis studies have been performed on this topic too.^22^,^23^ However, the results of the two meta- analysis studies were contrary to each other. Furthermore, several new prospective cohort studies on this topic had been published recently, and the results of these new cohort studies are inconsistent too.^24^,^25^ Therefore, it is necessary to perform an update meta-analysis to quantitatively summarize the association between coffee consumption and pancreatic cancer risk. Moreover in order to reduce the bias, we recruited prospective cohort studies only.

## METHODS

### Search strategy

We searched CBM (China Biology Medicine disc) and MEDLINE for studies of coffee consumption and pancreatic cancer risk up to June 2015. Key words searched were as follows: (pancreatic OR pancreas) AND (cancer OR tumor OR carcinoma) AND (coffee OR caffeine OR drinking OR beverages OR diet OR lifestyle). Moreover, we have scan reference lists of retrieved articles to search for additional studies. The language of the studies was limited to English or Chinese.

### Study selection

The inclusion criteria for the present meta-analysis were: (1) prospective cohort study design; (2) presented the consumption of coffee; and (3) provided the relative risk (RRs) (or odds rations or hazard ration) with their confidence intervals (CIs) (or data to calculate them). The exclusion criteria were: (1) case-control design; (2) data about coffee consumption was insufficient; (3) duplicate reports; (4) if multiple articles were from the same study population, only the one with largest sample or most information was included.

### Data extraction

Two authors (Ran and Wang) independently extracted all data and tabulated them, discrepancies were resolved by discussion. The following data from each eligible study was extracted: first author’s last name, year of publication, country, period of follow-up, number of participants, RRs of pancreatic cancer with corresponding 95% CIs for each level of coffee consumption, and variables adjusted for the statistical analysis.

### Statistical analysis

For all the included cohort studies, we computed overall RRs with 95% CIs for the highest *versus* lowest level of coffee consumption. Then subgroup analysis to evaluate the influence of geographic areas was performed too.

Statistical heterogeneity was investigated by *Q* test and *I*^2^ statistic. For the *Q* test, *P* < 0.10 was considered present heterogeneity. If the heterogeneity was statistically significant, a random effects model was conducted. Otherwise, a fixed effects model was used.

For the dose-response analysis of coffee consumption, the method proposed by Greenland *et al* was used to evaluate linear trends (study-specific slopes) from the correlated natural logs of the RRs through categories of coffee consumption.^26^ We only recruited those studies that showed the number of cases and person-years and RRs with variance estimates for at least three quantitative exposure categories. For each study, we assigned the midpoint of each exposure category as the dose corresponding, and the open-ended upper category was assumed to have the same amplitude as the previous category.

Finally, publication bias was evaluated by the Begg’s and Egger’s tests. Statistical analyses were performed with Stata (version 12.0; StataCorp, College Station, TX, USA).

## RESULTS

### Study characteristics

**Fig.1:**
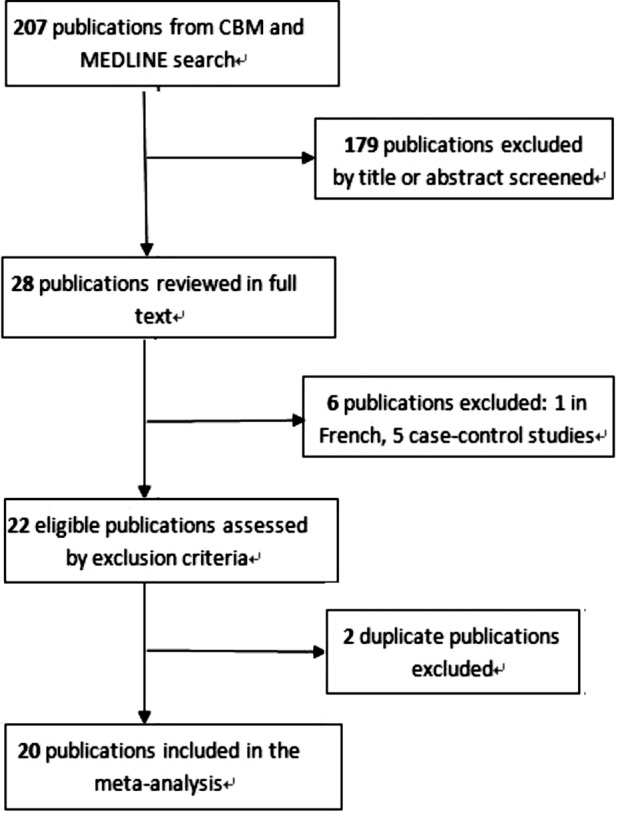
Flow diagram of selection of relevant publications.

A total of 20 cohort studies were identified in this meta-analysis, including 1341876 participants and 2872 cases of pancreatic cancer.^4^-^21^,^24^,^25^ The process of selecting studies is shown in Fig.1. Of the all eligible studies, nine studies were conducted in America (the United States),^4^-^10^,^12^-^14^ four studies in Asia (Japan),^16^,^18^,^19^,^21^ and seven studies in Europe (two each in Norway, Sweden, Finland, and one study was conducted in ten European countries by the European Prospective Investigation into Nutrition and Cancer cohort).^7^,^11^,^15^,^17^,^20^,^24^,^25^ The sample size varied from 412 to 477 312, and the number of pancreatic cancer cases ranged from 21 to 865 (Table-I).

**Table-I:** Characteristics of Studies of Coffee Consumption and Pancreatic cancer risk.

Study	Country	Study period	Cases/Subjects	Consumption categories	Relative risk (95% ci)	Adjustments
Nomura 1981 ^[4]^	America	1968-1981	28/8032	nondrinkers	1.00(reference)	age, smoking
				1-2 cup/d	2.74(0.61-12.36)	
				3-4 cup/d	1.80(0.36-8.89)	
				>5 cup/d	2.90(0.63-13.42)	
Whittemore 1983 ^[5]^	America	1966-1983	84/412	nondrinkers	1.00(reference)	age, college, class year
				drinkers	1.11(0.75-1.63)	
Snowdon 1984 ^[6]^	America	1960-1980	71/23912	<1 cup/d	1.00(reference)	age, sex
				1cup/d	1.7(0.9-3.3)	
				>2 cup/d	0.8(0.4-1.6)	
Jacobsen 1986 ^[7]^	Norway	1967-1978	63/16555	≤2 cup/d	1.00(reference)	age, smoking, residence
				3-4 cup/d	1.22(0.23-2.20)	
				5-6 cup/d	0.53(0.24-1.19)	
				≥7 cup/d	0.62(0.23-1.68)	
Hiatt 1988 ^[8]^	America	1978-1984	48/122894	nondrinkers	1.00(reference)	age, smoking, tea, alcohol, ethic, blood glucose
				<1 cup/d	0.8(0.3-2.6)	
				1-3 cup/d	0.9(0.4-2.1)	
				>4 cup/d	0.7(0.2-1.9)	
Mills 1988 ^[9]^	America	1976-1983	40/34000	Never	1.00(reference)	age, sex
				< Daily	0.65(0.22-1.89)	
				≥ Daily	0.71(0.34-1.48)	
Zheng 1993 ^[10]^	America	1966-1986	56/17633	<3 cup/d	1.00(reference)	age, smoking, alcohol
				3-4 cup/d	0.6(0.3-1.2)	
				5-6 cup/d	0.7(0.4-1.6)	
				>7 cup/d	0.9(0.3-2.4)	
Stensvold 1994^[11]^	Norway	1977-1988	41/42973	≤2 cup/d	1.00(reference)	age, smoking, country of residence
				3-4 cup/d	2.58(0.58-23.44)	
				5-6 cup/d	2.80(0.65-25.27)	
				≥7 cup/d	2.32(0.51-21.58)	
Shibata 1994 ^[12]^	America	1981-1990	63/13979	<1 cup/d	1.00(reference)	age, sex, smoking
				1cup/d	1.82(0.75-4.43)	
				2-3 cup/d	1.67(0.74-3.77)	
				≥4 cup/d	0.88(0.28-2.80)	
Harnack 1997 ^[13]^	America	1986-1994	66/33976	≤7cup/week	1.00(reference)	age, smoking
				8-17.5cup/week	1.91(0.92-4.00)	
				≥17.5cup/week	2.15(1.08-4.30)	
Michaud 2001^[14]^	America	1980-1998	288/136593	nondrinkers	1.00(reference)	age, smoking, body mass index, diabetes mellitus, history of cholecystectomy
				<1 cup/d	0.94(0.65-1.36)	
				1 cup/d	0.60(0.38-0.94)	
				2-3 cup/d	0.88(0.65-1.21)	
				>3 cup/d	0.62(0.27-1.43)	
Isaksson 2002 ^[15]^	Sweden	1961-1997	131/21884	0-2 cup/d	1.00(reference)	age, sex, smoking
				3-6 cup/d	0.91(0.60-1.38)	
				>7 cup/d	0.39(0.17-0.89)	
Lin 2002 ^[16]^	Japan	1988-1997	225/99527	nondrinkers	1.00(reference)	age, smoking
				1-2 cup/m	0.78(0.46-1.26)	
				1-4 cup/w	0.55(0.34-0.86)	
				1 cup/d	0.55(0.30-0.95)	
				2-3 cup/d	0.39(0.20-0.71)	
				≥4 cup/d	1.26(0.45-2.91)	
Stolzenbeng-Solomon 2002 ^[17]^	Filand	1985-1997	163/27111	reference category	1.00(reference)	age, smoking
				low	1.48(0.89-2.46)	
				moderately low	1.12(0.61-2.03)	
				moderately high	1.72(1.01-2.86)	
				high	0.95(0.54-1.68)	
Khan 2004 ^[18]^	Japan	1984-2002	25/3155	nondrinkers	1.00(reference)	age, smoking, education
				drinkers	0.38(0.01-1.05)	
Luo 2007 ^[19]^	Japan	1990-2003	233/102137	rarely	1.00(reference)	age, sex, smoking, body mass index, tea, alcohol, diabetes mellitus
				1-2 cup/w	1.0(0.7-1.4)	
				3-4 cup/w	1.1(0.7-1.7)	
				1-2 cup/d	0.9(0.6-1.3)	
				≥3 cup/d	0.8(0.4-1.3)	
Nilsson 2010 ^[20]^	Sweden	1992-2007	74/61569	<1 cup/d	1.00(reference)	age, sex, education, smoking, body mass index, physical activity
				1-3 cup/d	1.18(0.47-3.02)	
				≥4 cup/d	1.50(0.57-3.92)	
Nakamura 2011 ^[21]^	Japan	1992-1999	52/30826	nondrinkers	1.00(reference)	
				low	0.44(0.21-0.82)	
				high	0.33(0.15-0.69)	
Bhoo-Pathy 2013 ^[24]^	Europe	1992-2000	865/477312	low	1.00(reference)	age, sex, high, weight, education, smoking, body mass index, diabetes mellitus, physical activity
				nondrinkers	1.09(0.80-1.50)	
				moderately low	1.11(0.92-1.31)	
				moderately high	0.99(0.81-1.21)	
				high	1.07(0.86-1.33)	
Bidel 2013 ^[25]^	Finland	1972-2006	235/60041	nondrinkers	1.00(reference)	age, sex, alcohol, tea, study year, education, smoking, body mass index, diabetes mellitus, physical activity
				1-2 cup/d	0.86(0.42-1.74)	
				3-4 cup/w	0.86(0.45-1.64)	
				5-6 cup/w	0.78(0.41-1.47)	
				7-9 cup/w	0.92(0.46-1.83)	
				≥10 cup/d	0.82(0.38-1.76)	

### Highest versus lowest drinking category

As various measurement units for coffee consumption were used in the included studies, we just considered to compare the highest coffee consumption category with lowest coffee consumption category, and take the latter as the reference category.

**Fig.2:**
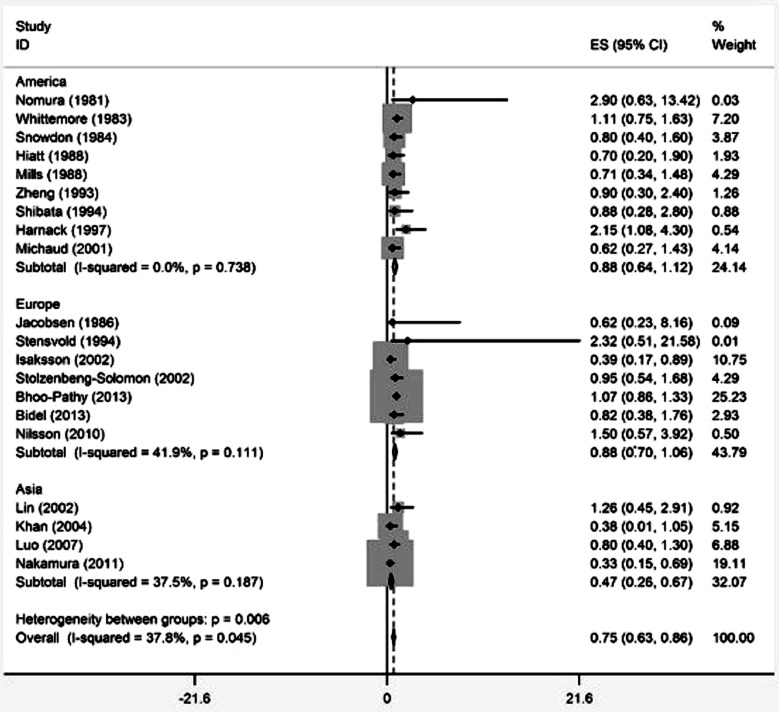
Forest plot (random-effects model) of coffee consumption (highest versus lowest category) and pancreatic cancer risk.

**Fig.3:**
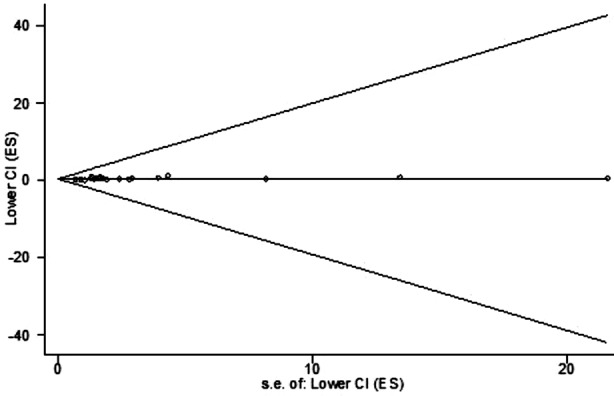
Begg’s funnel plot for publication bias.

The overall RR for highest coffee consumption *versus* lowest coffee consumption was 0.75 (95%CI, 0.63-0.86). Statistic significant heterogeneity was found among these studies (*I*^2^ =37.8%, *P* for heterogeneity =0.045) (Fig.2). Neither Egger’s test (*P* for bias =0.436) nor Begg’ test (*P* for bias =0.078) indicated a significant publication bias (Fig.3).

### Subgroup analysis on geographic areas

Nine studies had been conducted in the America, analysis of the nine studies showed that there was no association between coffee consumption and pancreatic cancer risk (RR, 0.88; 95%CI, 0.64-1.12; *P* for heterogeneity =0.738). And similar result was found in the seven studies in Europe (RR, 0.88; 95%CI, 0.70-1.06; *P* for heterogeneity =0.111). In contrast, when the four studies in Asia were pooled, coffee consumption was associated with a reduced pancreatic cancer risk (RR, 0.88; 95%CI, 0.70-1.06; *P* for heterogeneity =0.187) (Fig.2).

### Dose-response meta-analysis

Nine studies were included in the dose-response analysis for the relationship between coffee consumption and pancreatic cancer risk.^10^-^14^,^16^,^19^,^20^,^25^ The pooled RR for an increment of one cup/day of coffee consumption was 0.99 (95%CI, 0.96-1.03), without statistically significant. The Goodness-of-fit indicated no significant heterogeneity among these studies (*Q* = 35.29; *p* = 0.23).

## DISCUSSION

Up to now, more and more evidences, which is provided by epidemiological studies have demonstrated the inverse association between coffee consumption and some cancer risk, such as breast cancer, prostate cancer, liver cancer, and colorectal cancer.^27^-^31^ However the relationship between coffee consumption and pancreatic cancer risk had a series of inconsistent result. In 2011, a meta-analysis based on fourteen cohort studies showed a significant association between coffee consumption and reduced pancreatic cancer risk (RR, 0.68; 95%CI, 0.51-0.84).^22^ After that, Turati *et al* performed another meta-analysis with 37 case-control studies and 17 cohort studies, the result suggested non-significant association between coffee consumption and the risk of pancreatic cancer (RR, 1.13; 95%CI, 0.99-1.29).^23^ Specially, our update meta-analysis, which is based on 20 cohort studies, supported the protective effect of high coffee consumption for pancreatic cancer risk (RR, 0.75; 95%CI, 0.63-0.86).

In our meta-analysis, when the 20 studies were pooled, high coffee consumption was associated with a reduced pancreatic cancer risk, but the results of subgroup, which stratify by geographic area, were diverse. Studies in America and Europe showed a non-significant association between coffee consumption for pancreatic cancer risk. In contrast, studies in Asia revealed a significant inverse association between coffee consumption and pancreatic cancer risk. The diverse results among subgroup analysis may be owing to different race and environment.

Coffee interfere the cancerous process with different stage. The molecular mechanisms for anticancer effects of coffee compounds are as follows: (1) The antioxidant of coffee may reduce reactive oxygen species (ROS), that can induce DNA damage provoked.^32^ (2) Coffee can enhance endogenous defense systems by inducing a complex of nuclear clear factor erythroid-2-like 2 factor (Nrf2), and the cafestol of the coffee can increase the endogenous antioxidant too.^33^,^34^ (3) Coffee’s chemopreventive effect can induce DNA repair capacity. In vitro experiment suggested that cafestol and kahweol decreased 50% genotoxicity of human-derived hepatoma cells. 35 (4) Coffee consumption can decreased inflammation marker, such as the level of IL-18, c-reactive protein and E- selectin. Several compounds of coffee can also inhibit the activation of nuclear factor kappa B (NF-κB), that is the key transcription factor of inflammatory process.^36^,^37^ (5) Experiment showed that coffee component cafestol, kahweol, and caffeine can induce apoptosis. 38 These molecular mechanisms could well explain our findings in our meta-analysis that high coffee consumption with a decreased pancreatic cancer risk.

Several limitations of our meta-analysis should be discussed. First, we could not obtain enough information to calculate the adjusted RR in some studies, so we just combined unadjusted RRs. This could have influence on the quality of the meta-analysis. Second, as all cohort studies, the potential bias could not be completely avoided. Third, different classification coffee consumption among studies may contribute to the heterogeneity when pooled analysis. Finally, the different measurement units, brewing method, and coffee type may be the cause of heterogeneity too.

In summary, the present meta-analysis suggested that high coffee consumption is associated with a reduced pancreatic cancer risk. However, the result should be accepted with caution, due to the potential confounder and bias could not be completely excluded. Further well designed studies are needed to confirm the finding.
